# Matrix Metalloproteinase 9 Production by Monocytes is Enhanced by TNF and Participates in the Pathology of Human Cutaneous Leishmaniasis

**DOI:** 10.1371/journal.pntd.0003282

**Published:** 2014-11-13

**Authors:** Taís M. Campos, Sara T. Passos, Fernanda O. Novais, Daniel P. Beiting, Rúbia S. Costa, Adriano Queiroz, David Mosser, Phillip Scott, Edgar M. Carvalho, Lucas P. Carvalho

**Affiliations:** 1 Serviço de Imunologia, Universidade Federal da Bahia, Salvador, Bahia, Brazil; 2 Instituto Nacional de Ciências e Tecnologia – Doenças Tropicais, Salvador, Bahia, Brazil; 3 Department of Pathobiology, University of Pennsylvania, Philadelphia, Pennsylvania, United States of America; 4 Department of Cell Biology and Molecular Genetics, University of Maryland, College Park, Maryland, United States of America; 5 Instituto de Ciências da Saúde, Universidade Federal da Bahia, Salvador, Bahia, Brazil; University of Notre Dame, United States of America

## Abstract

**Introduction:**

Cutaneous leishmaniasis (CL) due to *L.braziliensis* infection is characterized by a strong inflammatory response with high levels of TNF and ulcer development. Less attention has been given to the role of mononuclear phagocytes to this process. Monocytes constitute a heterogeneous population subdivided into classical, intermediate and non-classical, and are known to migrate to inflammatory sites and secrete inflammatory mediators. TNF participates in the induction of matrix metalloproteinases (MMPs). MMP-9 is an enzyme that degrades basal membrane and its activity is controlled by the tissue inhibitor of metalloproteinase.

**Methods:**

Mononuclear cells were obtained from *ex-vivo* labeling sub-populations of monocytes and MMP-9, and the frequency was determined by flow cytometry. Culture was performed during 72 hours, stimulating the cells with SLA, levels of MMP-9 and TIMP-1 in the supernatants were determined by ELISA.

**Results:**

We observed that cells from CL lesions secrete high amounts of MMP-9 when compared to healthy subjects. Although MMP-9 was produced by monocytes, non-classical ones were the main source of this enzyme. We also observed that TNF produced in high level during CL contributes to MMP-9 production.

**Conclusions:**

These observations emphasize the role of monocytes, TNF and MMP-9 in the pathogenesis of *L. braziliensis* infection.

## Author Summary

To examine the participation of MMP-9 in the pathogenesis of *L. braziliensis* infection, we realized a cross-sectional study with CL patients in an early phase of the disease or with a classical ulcer, and healthy controls. We evaluated the frequency of MMP-9 in monocyte subsets and its mechanism of production. Our results showed that monocytes were the major cells producing MMP-9. The MMP-9 production by CL patients was presented in higher levels when compared with healthy subjects and early cutaneous leishmaniasis (ECL) patients, and the levels of MMP-9 inhibitor, TIMP-1, were lower in CL patients when compared to healthy subjects. The production of MMP-9 was enhanced by TNF, a cytokine associated with tissue damage in CL patients. Thus, therapeutic modulation of MMP-9 may be a useful approach for improving disease outcome in *L. braziliensis* patients.

## Introduction

Human cutaneous leishmaniasis (CL) caused by *Leishmania braziliensis* infection is characterized by the presence of one or more ulcerated lesions with raised borders and few parasites [1]. Early after infection, most patients develop lymphadenopathy, followed by the appearance of a papule at the bite site, which subsequently becomes an ulcerated lesion. These lesions are composed of a robust inflammatory infiltrate including the presence of T and B lymphocytes, mononuclear phagocytes and plasma cells [2]. It is well known that both CD4+ and CD8+ T cells have important roles in the control of *Leishmania* parasites replication [3]. During *L. braziliensis* infection these cells are activated and TNF and IFN-γ are produced in high levels both by peripheral blood mononuclear cells (PBMC) and at lesion site of CL patients. However, this response can also lead to tissue damage and development of the ulcer [4].

In contrast to the role of T cells in the pathogenesis of *L. braziliensis* infection less attention has been given to the contribution of mononuclear phagocytes to the inflammatory process and tissue damage observed in CL. In spite of the presence of tissue resident mononuclear phagocytes, circulating monocytes migrate to the infection site. In the tissue, they can differentiate into macrophages and dendritic cells (DCs), which are the main cell types parasitized, by *Leishmania*. It was recently proposed that in humans circulating monocytes are a heterogeneous population based on the surface expression of CD14 and CD16 [5]. The monocyte subsets are subdivided into classical (CD14++CD16−), intermediate (CD14++CD16+) and non-classical (CD14+CD16++) [5], [6]. It has been shown that CD16+ monocytes are able to produce high levels of TNF and increased frequency of these monocytes is associated with the immunopathogenesis of inflammatory diseases, such as arthritis and sepsis [7], [8], [9].

TNF is an inflammatory cytokine produced in high levels in CL patients [4]. TNF participates in the inflammatory process through the induction of nitric oxide, necrosis, citotoxicity and expression of matrix metalloproteinases (MMPs) [10], [11], [12]. MMPs are zinc-dependent enzymes that degrade extracellular matrix proteins and are functionally classified according to the specificities of the substrates they degrade [13]. MMP-9 is involved in degradation of collagen type IV, the major component of basal membrane present at skin [14]. While MMPs are necessary for successful eradication of infection by stimulating the migration of effector cells to the inflammatory site, high production of these molecules may induce pathology [15]. An imbalanced production of MMPs and its natural regulator, tissue inhibitor of metalloproteinases (TIMPs) occurs in a variety of diseases where tissue damage occurs [16], [17], [18]. *L. braziliensis*-infected macrophages secrete MMP-9, and its production is increased in patients with mucosal leishmaniasis, the more severe form of *L. braziliensis* infection [19]. Thus, while the factors that induce the breakdown of the basal membrane leading to the development of skin ulcer in leishmaniasis are not yet elucidated, we hypothesize that the lesion formation is due to breakdown dysregulation of the basal membrane caused by imbalance in levels of MMP-9 and TIMP-1, together with other factors, such as cell recruitment and edema, [20].

In the present study we examined the participation of MMP-9 in the pathogenesis of *L. braziliensis* infection. CL patients in an early phase of the disease or with a classical ulcer were evaluated. Initially, we showed that MMP-9 genes were highly expressed in the CL skin lesions. We then evaluated the production of MMP-9 and its inhibitor, TIMP-1, and identified an imbalance between MMP-9 and TIMP-1 ratio in CL patients. Monocytes were the main source of the enzyme. Finally, we found that high levels of TNF produced during the disease contribute to the up-regulation of MMP-9 synthesis in CL.

## Materials and Methods

### Patients

Participants of the present study includes 19 early cutaneous leishmaniasis (ECL) and 85 CL patients from the *L. braziliensis* transmission area of Corte de Pedra, Bahia state, Brazil, and 29 healthy subjects (HS) living in areas not exposure to *Leishmania*. ECL patients were characterized by the presence of a lymphadenopathy or lymphadenopathy accompanied by a papule or an exoulcerative lesion. Diagnosis for CL was performed by a positive parasite culture or PCR as previously described [21]. All patients were evaluated before treatment.

### Microarray-based expression profiling of human lesions

**Figure 1:**
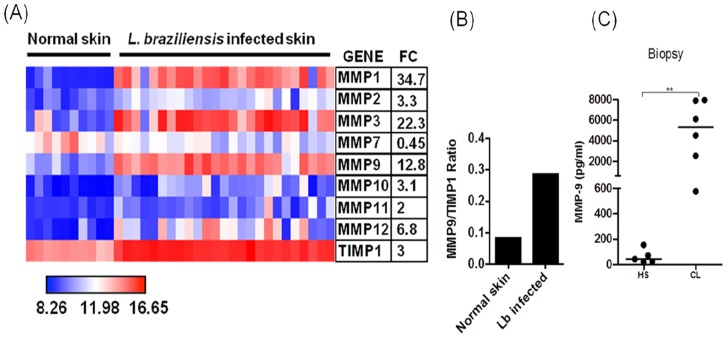
Lesions of CL patients produce MMP-9. *A*, Heatmap showing expression of MMPs and TIMP-1 from microarray profile. Biopsies from CL patients (*n* = 26) and normal skin (*n* = 10) were obtained and unbiased microarray was performed on biopsies mRNA. Average fold change (FC) for each gene in lesion samples relative to normal skin controls is shown. *B*, Ratio between MMP-9 and TIMP-1 genes expression. Biopsies from CL patients (*n* = 26) and normal skin (*n* = 10) were obtained and unbiased microarray was performed on biopsies mRNA. *C*, MMP-9 levels in biopsies culture supernatants from CL patients (*n = 6*) and healthy subjects (HS) (*n = 5*), determined by ELISA after the biopsies been cultured for 12 h in absence of stimuli. **p<0.005.

For whole genome expression microarray, lesion biopsies preserved in RNAlater (Qiagen) were homogenized using a rotor-stator and RNA was isolated using the RNeasy Plus kit (Qiagen). Biotin-labeled complementary RNA (cRNA) was generated using the Illumina TotalPrep RNA amplification kit (Ambion). RNA and cRNA quality were assessed on a BioAnalyzer (Agilent). Illumina HumanHT-12 version 4 expression beadchips were hybridized with cRNA from 26 *L. braziliensis* lesion biopsies and 10 biopses collected from uninfected donors. Arrays were scanned using a BeadStation 500GX and raw image files were processed using GenomeStudio v1.8 software (Illumina). Data was variance stabilized, robust-spline normalized, and quality control analysis carried out using the Lumi package [22] in Bioconductor/R. Differential expression analysis of the data using linear models and empirical Bayes methods [23] was carried out using the Limma package [24]. Data was deposited on the Gene Expression Omnibus (GEO) database for public access (GSE#GSE43880). Heat map tools available on GenePattern [25] were used to graphically display differentially regulated genes in Figure 1A.

### Soluble *Leishmania* Antigen (SLA) preparation

SLA was prepared with an isolate of *L. braziliensis* as previously described [26]. Briefly, promastigotes ressuspended in lysis solution (Tris, HCL, EDTA and leupeptin) were immersed in liquid nitrogen, and thawed at 37°C. After freezer-thaw procedure, they were sonicated and the disrupted parasites were centrifuged at 14,000G. The supernatant was filtered and assayed for protein concentration. SLA was used at a concentration of (5 µg/ml).

### Flow cytometry

Flow cytometry was performed as previously described [27]. Briefly, PBMC were ressuspended in saline and adjusted to a concentration of 0.5×10^6^ cells/ml. For *ex-vivo*, cell surface staining, we incubated cells with monoclonal antibodies anti-CD4 (APC), anti-CD8 (PE), anti-CD14 (APC) and anti-CD16 (PE) (BD bioscience), for 15 minutes, washed by centrifugation twice and fixed with 2% paraformaldehyde. For intracellular staining, cells were ressuspended in Perm/Wash (BD Cytofix/Cytoperm Plus – BD bioscience) for 15 minutes and intracellular labeling was performed using monoclonal antibody anti-MMP-9 (FITC) (BD bioscience) for 30 minutes. Cells were acquired on a FACS Canto II and analysis was done using Flowjo software (Tristar).

### Cultures of peripheral blood mononuclear cells and biopsies

Peripheral blood mononuclear cells (PBMC) were isolated from heparinized venous blood by Ficoll-Hypaque (GE Healthcare Bio-Sciences AB, Sweden) gradient centrifugation. After washing three times in 0.9% NaCl, PBMC were adjusted to 3×10^6^ cells/ml in 1 ml of RPMI-1640 (Gibco Laboratories, Grand Island, NY, USA) supplemented with 10% fetal bovine serum (FBS) (Gibco Laboratories, South America Invitrogen), 10 IU/ml penicillin and 100 µg/ml streptomycin. Cells were placed on 24 wells plates and incubated for 24 or 72 hours in the presence or absence of SLA (5 µg/ml) or recombinant TNF (5 ng/ml), or anti-TNF antibody (10 µg/ml) (R&D systems, Minneapolis, MN), as indicated in figures.

Biopsies from *L. braziliensis* patients and HS were cultured in complete RPMI media without stimuli. Tissue from CL patients and HS were cultured in RPMI for 12 hours. Supernatants from PBMC and biopsies were collected and stored at −70°C. The Levels of MMP-9, TIMP-1 (BD Biosciences, San Diego, CA, USA) and TNF (R&D Systems, Minneapolis, MN) were measured by ELISA according to the manufactures instructions. The results are expressed in pg/ml.

### Statistical analysis

Mann-Whitney was used to compare HS and CL groups; Kruskal-Wallis test (nonparametric test) was used to compare the ECL, CL and HS groups; Wilcoxon matched pair test (paired and nonparametric *t* test) was used to analyze PBMC cultures in different conditions within the same group of individuals. Comparisons were considered statistically significant when p<0.05. All *p* values represented are two-sided.

### Ethical considerations

This work was approved by the Ethics and Research Committee from Federal University of Bahia. All subjects provided witted informed consent; in case of illiterate subjects, a thumb print plus signature of an independent witness were used.

## Results

### MMP-9 is produced at lesion site from CL patients

MMPs are enzymes that degrade extracellular matrix and in high levels MMPs may cause tissue damage [15]. Exaggerated inflammatory responses leads to tissue damage and ulcer development in CL [4]. To determine whether MMPs were expressed in CL lesions, we performed a whole genome expression profile from lesions of CL patients (n = 26) and compared that to normal skin (n = 10). The results showed that several MMPs, including MMP-9, had increased expression over normal skin (Figure 1A). We also assessed the expression of TIMP-1 in the skin and although TIMP-1 expression was increased in *L. braziliensis* infected skin over healthy skin, the ratio MMP-9/TIMP-1 expression was higher in CL skin when compared to normal tissue (Figure 1A and B). Because MMP-9 is known to be involved in basal membrane disruption, a process that precedes ulcer development in CL, we tested whether MMP-9 protein was produced in the lesion of CL patients and healthy subjects. To address that we cultured whole biopsies in the absence of stimulus for 12 hours and determined MMP-9 levels on culture supernatants by ELISA. Cells from biopsies from CL patients produced significantly more MMP-9 than those from healthy subjects (HS) (Figure 1C). These results show an imbalance between MMP-9 and TIMP-1 expression, suggesting that MMP-9 participate in lesion development in CL.

### Imbalanced production of MMP-9 and TIMP-1 in CL patients

**Figure 2:**
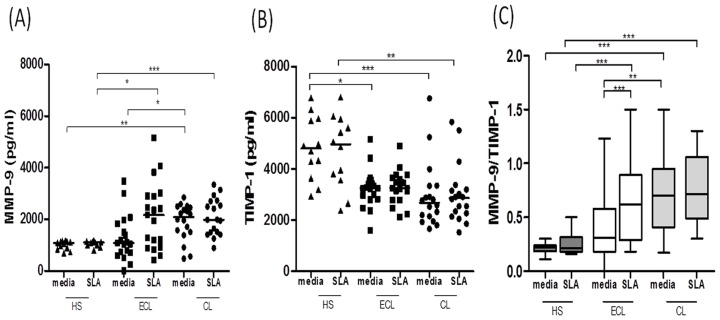
CL patients have high levels of MMP-9 and low levels of TIMP-1 when compared with healthy subjects. PBMC were obtained from healthy subjects (HS) (*n = 12*), early CL (ECL) (*n = 19*) and CL patients (*n = 18*), and levels of MMP-9 (*A*) and TIMP-1 (*B*), determined by ELISA in culture supernatants of PBMCs upon stimulation with soluble *Leishmania* antigen (SLA) (5 µg/ml) for 72 hours. *C*, Ratio between MMP-9 and TIMP-1 in culture supernatants of PBMCs upon stimulation with SLA. The box-and-wickers plot shows the minimum, first quartile, median, third quartile, and maximum values of a set of data. The wickers represent error bars and those represent minimum and maximum values, indicating variability outside the upper and lower quartiles. Data shown from a representative experiment of five performances. *p<0.05; **p<0.005; ***p<0.0005.

TIMPs are glycoproteins that inhibit MMPs and TIMP-1 specifically regulates the activity of MMP-9. Therefore, our next step was to determine the production MMP-9 and TIMP-1 by PBMC from HS and CL patients in the early and late phase of the disease. To do so, PBMC were stimulated in vitro with SLA or left untreated (media) for 72 hours and the production of MMP-9 and TIMP-1 were determined in the supernatants by ELISA. Our results show that CL patients produced high levels of MMP-9 and low levels of TIMP-1 when compared to HS (Figure 2A and 2B). Interestingly, PBMCs from CL patients produced high levels of MMP-9 even in the absence of stimulus, indicating that those cells are prone to produced MMP-9 when still in CL patient. A change in the ratio of MMPs and TIMPs may cause excessive degradation of the extracellular matrix and consequently tissue damage [28]. Therefore, we next sought to determine the ratio between MMP-9 production and TIMP-1. The high ratio MMP-9/TIMP-1 in cultures from CL individuals indicated an imbalance in the production of these enzymes in *L. braziliensis* infected individuals when compared to HS (Figure 2C).

### MMP-9 is mainly produced by monocytes from CL patients

**Figure 3:**
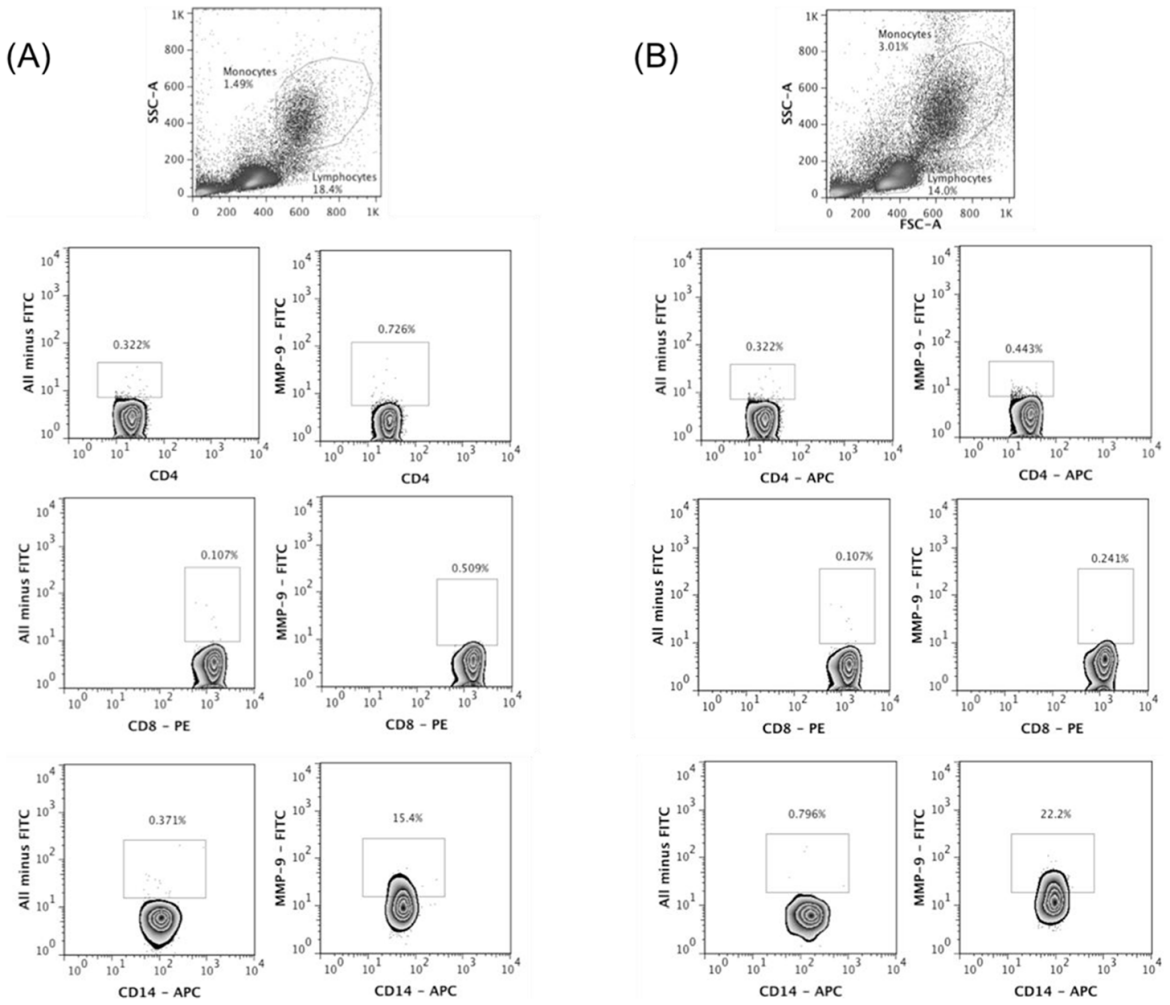
Monocytes are important source of MMP-9 in CL. PBMC were obtained from healthy subjects (HS) and CL patients and intracellular labeling to MMP-9 was performed *ex-vivo*. The frequency of CD4+, CD8+ and CD14+ cells positive to MMP-9 was determined by flow cytometry. The gate strategy was done using all minus one staining. *A*, representative plots from HS (*n* = 5). *B*, representative plots from CL patients (*n* = 5).

It has been documented that leucocytes are the main source of MMP-9 [29], [30] and our results show that PBMCs from CL patients secrete high levels of MMP-9 in response to SLA. To further characterize the source of MMP-9 in CL patients, using flow cytometry we determined the *ex-vivo* production of MMP-9 by CD4+ and CD8+ T cells, and monocytes based on CD14 expression. Our results show that monocytes are the main source of this enzyme in HS and patients with CL (Figure 3A and B).

**Figure 4:**
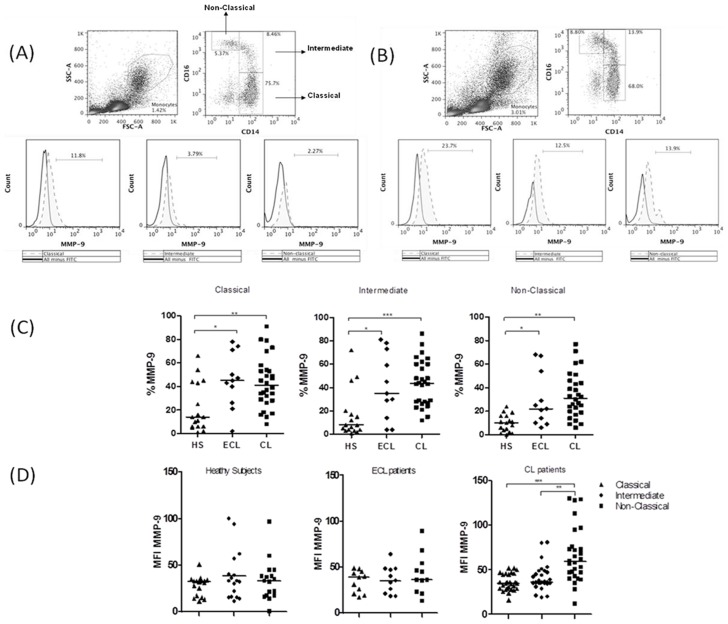
Monocyte subsets from CL patients produce MMP-9. PBMC were obtained from healthy subjects (HS) (*n = 17*), early CL (ECL) (*n = 11*) and CL patients (*n = 28*), and staining to CD14, CD16 and MMP-9 was performed. Frequency of monocyte subsets producing MMP-9 was determined *ex-vivo* by intracellular staining. Gate strategy to assess monocyte subsets based on CD14 and CD16 expression and frequency of MMP-9 in each subset of monocytes in HS (*A*) and CL patients (*B*). The gate strategy was done based on all minus one staining. *C*, Frequency of MMP-9-producing monocyte subsets from HS, ECL and CL patients. *D*, Mean fluorescence intensity (MFI) of MMP-9 positive cells in monocyte subsets from HS, ECL and CL patients. Data shown from a representative experiment of four performances. *p<0.05; **p<0.005; ***p<0.0005.

Recently, three monocyte subsets have been described based on the expression of CD14 and CD16, subdividing them into classical, intermediate and non-classical monocytes [5]. In our study the gate strategy to access the monocyte subsets was defined based on CD14 and CD16 expression. To evaluate the contribution of monocyte subsets to MMP-9 production we performed intracellular *ex-vivo* staining of PBMC from patients with ECL, CL and HS which were then analyzed by flow cytometry. The frequency of MMP-9 in each subsets of monocyte was performed using an isotype control (Figure 4A and B). Our data shows that monocytes from patients with ECL and CL express more MMP-9 than monocytes from HS and that non-classical monocytes (CD14+CD16++) are the major source of MMP-9 in patients with CL (Figure 4C and 4D).

### TNF enhances MMP-9 production in CL patients

**Figure 5:**
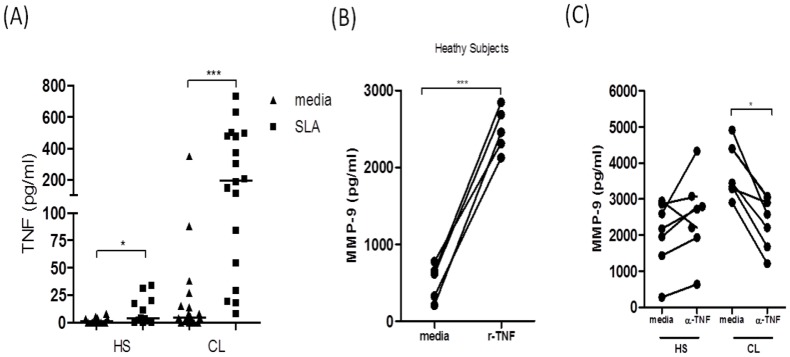
TNF enhances MMP-9 production in CL patients. *A*, PBMC from healthy subjects (HS) *(n = 12)* and CL patients (*n = 19)* were cultured in the presence or absence of SLA (5 µg/ml) for 72 hours, and TNF concentrations were determined by ELISA on supernatants. *B*, PBMC from HS (*n = 5*) were cultured in the presence or absence of recombinant TNF (5 ng/ml) for 24 hours and the levels of MMP-9 were determined by ELISA. *C*, PBMC from HS *(n = 6)* and CL patients *(n = 6)* was cultured in presence or absence of anti-TNF antibodies (10 µg/ml) for 24 hours. The levels of MMP-9 were determined by ELISA on supernatants. *p<0.05; ***p<0.0005.

TNF is an inflammatory cytokine that contributes to tissue damage by different mechanisms, including by induction of MMP-9 expression [31]. To determine if TNF production contributes to MMP-9 production in CL patients, we first measured TNF levels in supernatants of SLA-stimulated PBMC from HS and CL patients. As previously demonstrated [32], CL patients produced high levels of TNF in response to SLA (Figure 5A). In order to determine if the TNF produced had the ability to increase MMP-9 expression, we added recombinant TNF to PBMC cultures from HS and determined MMP-9 expression in the supernatants by ELISA. Our results show that there was a 5-fold increase in MMP-9 production in unstimulated PBMC cultures from healthy individuals upon exogenous addition of recombinant TNF (Figure 5B). Finally, we asked if TNF was playing a role in MMP-9 production in CL. In order to address this question, we added anti-TNF antibodies to PBMC cultures from HS and CL patients (Figure 5C) and we found that the blockage of TNF inhibits the production of MMP-9 in CL patients. Altogether these data show that TNF, in part, regulates MMP-9 production in CL patients.

## Discussion

CL is characterized by a well-defined ulcer with raised borders that appears a few weeks after the transmission of the parasite by sandflies [33]. One of the first signs of the disease is lymphadenopathy followed by the development of a papular or an exoulcerative lesion at the bite site, 2–3 weeks later. Finally, 1–2 weeks later the classical ulcer is observed [33], [34]. The evaluation of the immune response during the phase in which patients has not yet developed the cutaneous ulcer is important to determine factors contributing to disease development. Our group has described the immune response in CL patients and discovered the presence of many cytokines and chemokines that contributes to the maintenance of inflammatory response in these individuals [35]. However, much less has been done to understand the immune response at the early stages of the disease. This knowledge is particularly important, as it may unravel the factors that contribute to disease severity and/or ulcer development. Here, we investigated the contribution of leucocytes to MMP-9 production in CL patients and for the first time, we show how monocytes contribute to the production of MMP-9 in CL.

MMPs mediate several physiological processes, such as cell migration, extracellular matrix degradation and tissue remodeling. Our *in situ* gene expression data shows that MMP-1, MMP-3 and MMP-9 expression were particularly increased in CL over healthy skin. While MMP-1 and MMP-3 are more associated with matrix remodeling,uncontrolled secretion of MMP-9 has been associated with pathological processes [28], [36], [37]. There are a few examples of a role for MMPs in the pathogenesis of *Leishmania* infection: in a murine model of *L. chagasi* infection, production of MMP-9 by macrophages was associated with tissue damage [38]; high levels of mRNA to MMP-2 were documented in ulcers of CL patients and in macrophages from mucosal leishmaniasis patients; and upon infection with *L. braziliensis* human macrophages increased secretion of MMP-9 [19]. As MMP-9 degrades type IV collagen, the main component of the basement membrane in the skin, and their activation causes excessive tissue damage by facilitating the migration of inflammatory cells to the infection site [28]. In the present work we found that the MMP-9 gene was expressed during disease when compared to normal skin and we were also able to detect high levels of this enzyme being secreted by cells obtained from *L. braziliensis* lesions. With these findings we hypothesize that MMP-9 plays a role in ulcer development during CL. Our results led us to determine the levels of MMP-9 produced by PBMC. High levels of MMP-9 were observed in supernatants of PBMC from patients when compared with those from healthy subjects. Because exaggerated production of MMPs are associated with immunopathology [15], TIMPs, the natural inhibitors of these enzymes, play an important role in controlling MMPs production and activity. As expected, the levels of TIMP-1 were increased in healthy subjects when compared to CL patients, revealing an imbalance between MMP-9/TIMP-1 in CL individuals. MMP-9 activity was previously associated with development of mucosal leishmaniasis, the more inflammatory form of the disease with extensive tissue damage, and expression of MMPs were also correlated with therapeutic failure in CL [19], [39]. These data confirm the potential role for these enzymes in mediating immunopathology during leishmaniasis. Our documentation that patients in the pre-ulcerative phase of the disease produced lower levels of MMP-9 than patients with classical ulcer, raise the possibility that progression of the disease to the ulcerative phase may be associated with increasing in MMP-9 levels and, consequently, imbalance between MMP-9 and TIMP-1.

Evidence has been accumulated that activation of both CD4+ and CD8+ T cells are associated with tissue damage in CL [40], [41], [42]. On the other hand, less attention has been given to the role of mononuclear phagocytes in the pathogenesis of CL. Macrophages are the main reservoirs of the *Leishmania*, responsible for *Leishmania* parasite killing [43] but macrophages also contribute to the production of TNF-α and pro-inflammatory chemokines, which are associated with severe forms of the disease [44]. It was previously reported that different leukocytes contribute to deleterious MMPs secretion in infectious diseases [29], [30], [45], [46]. In a mouse model of toxoplasmosis, in addition to inflammatory monocytes and neutrophils, CD4+ and CD8+ T lymphocytes where also important source of MMP-8 and MMP-10 [47]. In malaria and tuberculosis MMP-9 was predominantly produced by activated monocytes [45], [46]. Here we show that in CL, although CD4+ and CD8+ T lymphocytes participate in the pathological immune response through the production of inflammatory mediators or cytolytic activity, monocytes were the main cells producing MMP-9. Neutrophils are another cell type known to produce high levels of MMP-9 in other inflammatory conditions [48], but since in CL the cellular infiltrate is predominantly mononuclear, neutrophils may not be a primary source of MMP9 in these patients [49].

Monocytes are subdivided in three subsets according to the expression of CD14 and CD16, and each subset has distinct roles during the infectious process. Classical monocytes (CD14++CD16−) have high phagocytic capacity [5], [6] and express more anti-microbicidal molecules, like reactive oxygen species, acting as an important line of defense against *L. braziliensis* (Novais and Nguyen et al., unpublished data). In contrast, intermediate monocytes (CD14++CD16+) express more MHC class II and act more effectively as antigen presenting cells than the other subsets of monocytes [5], [6]. Controversial studies have been published regarding the function of non-classical monocytes (CD14+CD16++) as some have reported that these cells produce more pro-inflammatory cytokines and are responsible for the recruitment of cells to the inflammatory sites, and others have documented a regulatory role for them [5], [6]. Although in this study all monocyte subsets produced MMP-9, the intensity of expression of this molecule was higher in non-classical monocytes from some individuals. It was previously shown that the expression of CD16 in monocytes is associated with high production of MMP-9 and increased frequency of CD16+ monocytes has been documented in CL patients [50] [51]. Thus, future studies will be performed to determine the ability of these cells to migrate into tissues to determine the contribution of different monocyte subsets to immunopathology at lesion site.

The production of MMP-9 is not spontaneous, being dependent on the interaction of cell to cell, cell to matrix and/or in response to cytokines [52]. As an important inflammatory cytokine TNF is involved in immune regulation and resistance to various microorganisms and exerts a variety of biological activities such as apoptosis, cytotoxicity and induction of MMPs [10], [11], [12]. We showed here that in CL TNF plays an important role in the regulation of MMP-9 since addition of exogenous TNF enhanced MMP-9 production in HS, whereas neutralization of this cytokine down-regulated MMP-9 synthesis in CL patients. Therefore we propose TNF as a major regulator of MMP-9 production during *L. braziliensis* infection. Our observations agree with what has been previously reported for other diseases [45], [46]. For example, in malaria, activation of MMP-9 was dependent upon the production of TNF and its activation was decrease by anti-TNF treatment [46].

This study brings evidence for the role of monocytes in the pathology associated with *L. braziliensis* infection and suggests that monocytes and MMP-9 may play key roles in tissue destruction. Specifically, the excessive production of TNF by monocytes observed in CL increases the production of MMP-9 leading to an imbalance between production of this enzyme and its inhibitor, TIMP-1. As a consequence, there is an excessive degradation of the basal membrane, migration of inflammatory cells to the site of infection and ulcer development. Thus, therapeutic modulation of MMP-9 may be a useful approach for improving disease outcome in *L. braziliensis* patients.
